# Navigating the Intrahepatic Cholestasis of Pregnancy: An Autobiographical Case Report

**DOI:** 10.7759/cureus.66770

**Published:** 2024-08-13

**Authors:** Barna Dam

**Affiliations:** 1 Department of Internal Medicine, Kumudini Women’s Medical College, Tangail, BGD

**Keywords:** autobiographical case report, gestational diabetes mellitus, intense pruritus, gestational disorder of the liver, intrahepatic cholestasis of pregnancy (icp)

## Abstract

Intrahepatic cholestasis of pregnancy (ICP) is a liver condition commonly occurring during pregnancy, with an unclear etiology. This condition not only causes significant discomfort for the mother due to severe itching but also poses serious risks to the fetus. Effective and timely management of ICP, including diagnosis, consistent monitoring, and treatment, is crucial to mitigate maternal discomfort and prevent fetal complications. The challenges in managing ICP include the absence of clear initial diagnostic criteria, delays in lab results, evolving treatment guidelines, and the financial burden of therapy. This case report shares the author's personal encounter with ICP, detailing the diagnosis, treatment pathway, impacts on the newborn, and the emotional journey during and after pregnancy. This report aims to enhance understanding and awareness of ICP, particularly among populations in the United States where the disease is less prevalent.

## Introduction

Intrahepatic cholestasis of pregnancy (ICP) is the most frequent liver condition linked to pregnancy, and its prevalence varies by ethnicity and geographic location [1]. ICP generally manifests in the third trimester [2] but can appear as early as the first trimester [3] or second trimester, as shown in this study. In the United States, the Latinx population experiences the highest incidence of ICP, significantly more (i.e., 10-100 times) than other ethnic groups [4], suggesting the need for further research across different ethnicities. The primary symptom of ICP is pruritus, which can range widely in severity, along with other symptoms like right upper quadrant pain, pale stools, and dark urine [2]. Diagnosis is typically confirmed through serum total bile acid testing, although there is no consistent link between the severity of pruritus and bile acid levels. Other laboratory values such as aspartate aminotransferase (AST), alanine transaminase (ALT), alkaline phosphatase (ALP), gamma-glutamyl transferase, and bilirubin might also be elevated. ICP poses considerable risks to the fetus, including the potential for stillbirth and the need for specialized neonatal care, highlighting the critical need for agreed diagnostic and management protocols to mitigate perinatal morbidity [5].

This case report narrates my personal diagnosis of ICP at 21 weeks, gestational diabetes mellitus (GDM) at 28 weeks, and the premature delivery of my baby at 35 weeks and six days, along with the emotional impact of these experiences. The relationship between ICP and GDM is complex and sometimes contradictory, with some studies noting a positive correlation [6] and other studies finding no correlation [7]. In presenting my story, I aim to enhance the understanding of ICP’s epidemiology, pathophysiology, clinical presentation, and management, as well as its long-term health implications. By sharing my experience, this report seeks to offer a candid view into the psychological experience of living with ICP and GDM, encouraging further dialogue and study of these conditions.

## Case presentation

At 21 weeks and three days into my pregnancy, I was awoken in the middle of the night by intense itching. Despite my efforts, the scratching persisted throughout the night. In the morning, I noticed that my thighs and feet were red and raw from the scratching. The itching was particularly severe on the palms of my hands and the soles of my feet. This was my first pregnancy at age 30, and until then, it had been smooth, with only a controlled hypothyroidism condition for the past four years. I have not changed my soap, cream, or detergents recently, nor do I have a history of allergies. Concerned, I contacted my OB-GYN's office first thing in the morning.

After hearing the symptoms, my doctor could see me for an urgent noon appointment the same day. My doctor saw me directly; she listened empathetically and took my complaint seriously. She requested serum bile acid levels to be tested along with other blood and liver function tests (LFTs). The blood was drawn on the same day (October 27, 2021). The blood and LFT results came back by 5 pm the same day, and the ALT and AST levels were 188 unit/L (reference range: 7 unit/L-52 unit/L) and 128 unit/L (reference range: 11 unit/L-39 unit/L), respectively. ALT and AST were almost four times higher than the normal range, but ALP was within the normal range, i.e., 96 unit/L (reference range: 50 unit/L-135 unit/L). My doctor immediately called me at around 6 pm and described the potential scenario with the LFT elevated. She also mentioned ICP as a likely diagnosis for my symptoms. She prescribed me ursodiol (300 mg capsule) and advised me to start immediately. She also referred me to Maternal and Fetal Medicine (MFM) for closure follow-up and treatment plans.

Over the next week, as I waited for the results from my bile acid tests, the itching persisted, intensifying to an eight out of 10. It spread across my entire body, disrupting my sleep significantly. I struggled to fall asleep and often awoke within an hour, finding myself scratching uncontrollably in my sleep. To distract myself, I immersed myself in my studies and dedicated considerable time to researching intrahepatic cholestasis of pregnancy (ICP). In addition, I joined an ICP-focused social media group for mothers. This community proved invaluable, offering insights into real-life experiences and sharing personal stories and coping strategies.

The MFM doctor saw me on October 29, 2021, and we discussed cholestasis, given that my LFTs were elevated, but confirmation is pending until receipt of bile acids results. The doctor mentioned that this would be very early for a diagnosis of cholestasis in pregnancy and might need to do further GI work-up if bile acids do not resolve after pregnancy. We also discussed antenatal testing and reviewed its limited efficacy in the detection of cardiotoxicity to the fetus from cholestasis and its limitations at this gestational age. The doctor mentioned that ICP is considered when the patient develops new onset pruritus without rash in the second half of pregnancy. It is commonly total body itching with severe pruritus on the palms and soles and worse at night. The cause is unknown, but at least some cases are related to gene mutations controlling the hepatocellular transport system. During the same visit, I had a follow-up ultrasound, which showed no abnormalities and a normal heart rate.

On November 2, 2021, I received confirmation from my doctor's office that I had ICP, with my total bile acid level measured at 20.33 µmol/L, significantly above the normal reference value of less than 10 µmol/L. I was prescribed ursodeoxycholic acid (ursodiol) 300 mg to be taken twice daily with meals. Following this diagnosis, I was instructed to arrange a follow-up appointment with the MFM office for ongoing monitoring and management of the condition. I scheduled this important follow-up for November 8, 2021. MFM doctor recommended weekly limited ultrasounds (until antenatal testing can begin), follow-up growth ultrasounds every four weeks, and beginning of twice weekly biophysical profiles (BPPs) at 28 weeks gestation. He also recommended blood workups for bile acids, AST, and ALT every two weeks. Because of my history of hypothyroidism (with treatment), the doctor recommended a follow-up ultrasound with fetal thyroid assessment at 28-30 weeks of gestation. On the day of this visit, I was 24w+2d gestation, and depending on the current lab values, doctors recommend delivery at 37 weeks.

Over the next six weeks, I was under regular monitoring (i.e., BPP, limited ultrasound, follow-up ultrasound, and LFT blood work every week). The baby’s growth and all other laboratory values and imaging were within normal limits. On December 6, 2021 (gestational age of 28w+2d), a one-hour glucose test was 178 mg/dL, higher than the standard 70-140 mg/dL range. The initial result was directed toward GDM; however, the doctor recommended performing a three-hour gestational glucose tolerance test (GTT) as a confirmatory test. On December 14, a three-hour GTT was performed, and one, two, and three hours. glucose values were 225 mg/dL (reference: 50-180), 236 mg/dL (reference: 50-155), and 106 mg/dL (reference: 50-140). Just before the three-hour test point, I vomited, and therefore, the three-hour test result was declared invalid. However, under the current guideline, if two of these three test points are positive, it indicates positive GDM. In my case, one- and two-hour glucose values were already positive, and therefore, I was diagnosed with GDM in addition to the ICP. The doctor recommended dietary modification and recording blood glucose values four times daily, including fasting glucose in the morning. Due to elevated blood glucose levels in the next two weeks, the doctor prescribed metformin 500 mg daily (30w+2d gestation).

**Figure 1:**
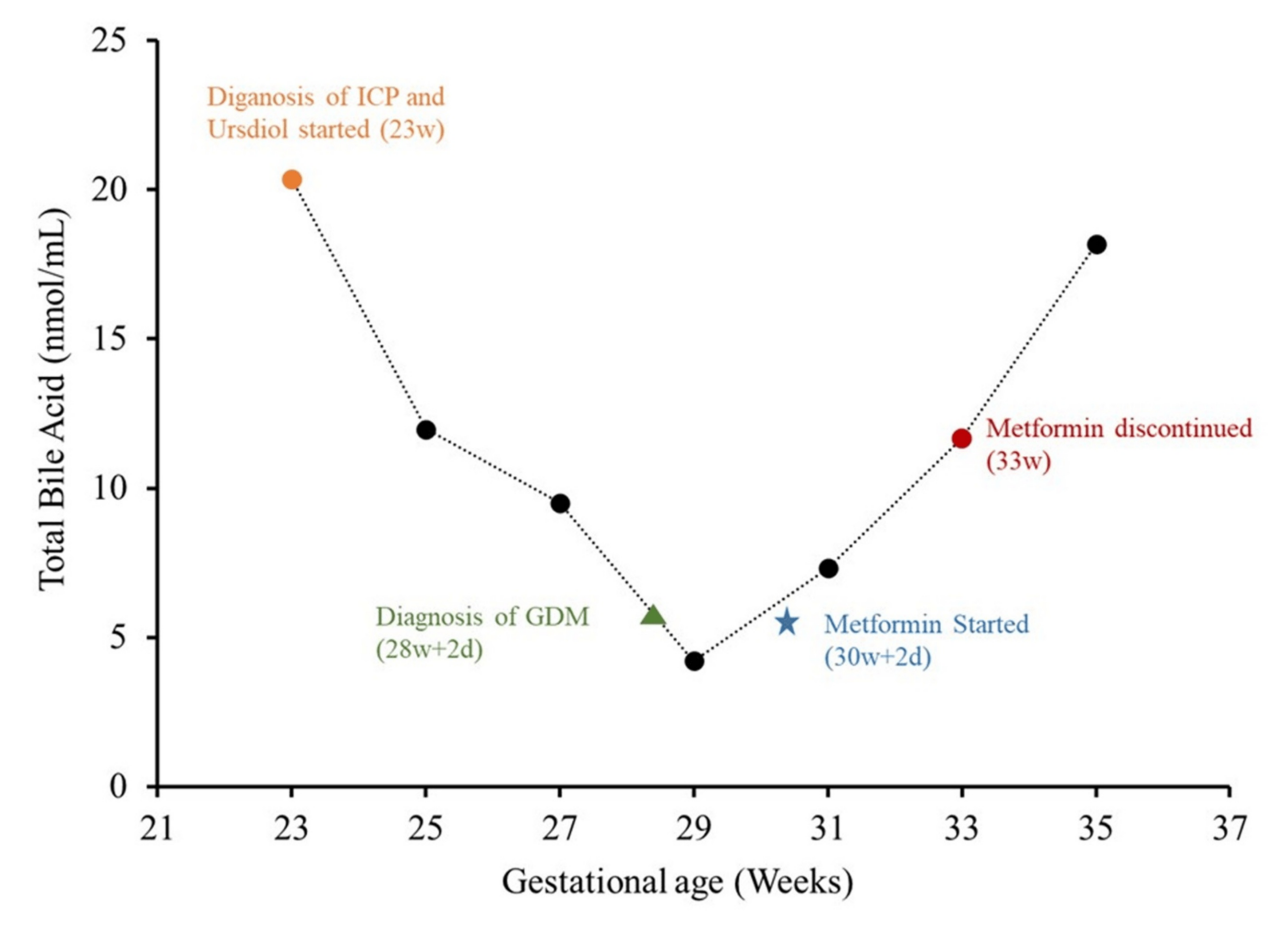
Timeline summary of significant incidents and total bile acid trend during pregnancy ICP = Intrahepatic cholestasis of pregnancy, GDM = gestational diabetes mellitus

My itching suddenly returned and increased over time as I started taking metformin. The ALT and AST also increased after taking metformin, and after two weeks (on January 13, 2022), the total bile acid concentration increased to 11.66 nmol/mL (reference: 10 nmol/mL). Doctors suspected that it might be due to taking metformin or due to cholestasis. The blood glucose levels were normal in recent weeks, so the doctor advised stopping metformin. However, the doctor also mentioned using a low insulin dose if needed. The ALT, AST, and total bile acid concentrations kept rising and reached 18.18 nmol/mL on January 26, 2022. The doctor recommended delivery at 36 weeks, and my induction of labor (IOL) was scheduled for January 29, 2022 (gestational age 35w+6d). On the day of IOL, the fetus was found to be in a transverse position during the initial ultrasound. Doctors discussed the situation, and alternatives between performing an external cephalic version (ECV) followed by vaginal delivery and cesarean section were discussed. They also discussed the risks, including fetal distress, abruption, and the need for emergency cesarean section in case of ECV. Risks of cesarean section include infection, bleeding, blood clots, scarring, injury to the fetus, heart attack, stroke, and death. After discussing it with my husband, I opted for a cesarean section. I had an uneventful operation (cesarean section), and my son was born just after midnight. He weighed 5 lb. 10 oz and was 1 ft. 8 inches tall. The neonatal intensive care unit (NICU) was never used for my son, and he was able to breathe effortlessly on his own since his birth. He received phototherapy for the next two days due to neonatal jaundice, otherwise uneventful. We were both discharged from hospital on day 5. A simplified timeline is illustrated in Figure 1.

## Discussion

Epidemiology of ICP

ICP is the most common pregnancy-related liver disease, with a worldwide variable incidence rate between 0.2% and 27.6% depending on ethnicity and geographical location, and it is primarily diagnosed in pregnant women in South America and northern Europe [1]. Lee et al. (2006) reported that the prevalence of ICP in California is 5.6%, more than 10-100 times the reported rate in the general population of the United States [4]. The risk of recurrence is up to two-thirds in subsequent pregnancies [8].

Pathophysiology of ICP

The pathophysiology of ICP remains insufficiently understood. Several risk factors are identified, such as a personal or family history of ICP, hepatitis-C infection, the use of oral contraceptives, multiple pregnancies, in-vitro fertilization, and seasonal variation: winter [5, 9-11]. Normally, cholesterol is used to synthesize bile acids in the liver, which are then stored in the gallbladder and released into the intestine to aid digestion [10]. In cases of ICP, however, bile acids accumulate in the liver, likely due to genetic alterations that affect bile acid elimination and lead to high serum bile acids [10].

These elevated bile acid levels in pregnant women can have detrimental effects on the fetus. Although the placenta typically acts as a barrier against harmful substances, including bile acids, this protective function is compromised in ICP [9]. As bile acid concentrations rise in the mother's bloodstream, they also increase in the fetus, potentially resulting in serious complications like meconium-stained amniotic fluid or stillbirth [9,11]. In addition, higher bile acid levels are believed to harm the placenta, although the specific changes and their impacts are poorly understood. Theories such as those proposed by Williamson et al. suggest that fetal distress could be caused by placental blood vessel constriction leading to oxygen deprivation or by bile acid-induced fetal arrhythmias resulting in cardiac arrest [12].

Diagnosis of ICP

ICP typically manifests with sudden, intense itching, especially on the palms and soles, which often intensifies at night. Although a rash is usually absent, some individuals may develop skin excoriations due to relentless scratching. The symptoms of ICP are generally confined to the pregnancy period and typically resolve after childbirth [13], which was also similar to my case.

The clinical diagnosis of ICP is supported by elevated serum total bile acids and transaminases. A total bile acid concentration above 10 nmol/mL and increased AST/ALT levels beyond the normal range indicate ICP. However, the diagnosis can be delayed because (1) symptoms such as pruritus and elevated LFT may appear before the increase in bile acids, and (2) specialized lab tests for bile acid quantification can take up to two weeks [13]. While LFT is not essential for diagnosing ICP, the presence of transaminitis can raise suspicions of the condition during the wait for bile acid results. Nonetheless, normal ALT and AST levels do not rule out ICP.

The fetal risk escalates with higher bile acid levels, with concentrations ≥40 nmol/mL indicating a significant risk [13]. Di Mascio et al. reported a 6.8% incidence of fetal and newborn mortality when bile acids reached ≥100 nmol/mL [14]. According to guidelines from the Society for Maternal-Fetal Medicine (SMFM), perinatal care providers should consider immediate delivery if bile acid levels are high and the pregnancy is in the late preterm or term stages [13]. Given that bile acid levels can fluctuate during pregnancy, retesting in cases of persistent or worsening symptoms is crucial to ensure accurate diagnosis and prevent mismanagement. Table 1 illustrates the variation in laboratory values, including LFT and bile acids, throughout my gestation.

**Table 1:** Laboratory values (LFT and bile acid) throughout gestation LFT = liver function tests, ALT = alanine transaminase,  AST = aspartate aminotransferase, NT = not tested, a = postpartum measurement.

Gestational age (weeks)	Laboratory test (reference value), unit						
	Alanine transaminase (ALT), (7–52), Units/L	Aspartate aminotransferase (AST), (11-39), Units/L	Total cholic acid (<5), nmol/mL	Chenodeooxycholic acid (<6), nmol/mL	Total deoxycholic acid (<6), nmol/mL	Total ursodeoxycholic acid (<2), nmol/mL	Total bile acid (<10) nmol/mL
23	188	128	9.45	7.59	2.77	0.52	20.33
25	44	34	1.94	1.99	0.21	7.84	11.98
27	57	45	0.95	1.61	0.89	6.06	9.51
29	45	32	0.72	0.55	0.17	2.78	4.22
31	298	144	1.4	1.32	0.67	3.92	7.32
33	262	144	1.43	1.62	0.79	7.82	11.66
34	148	76	NT	NT	NT	NT	NT
35	104	64	2.96	2.67	1.59	10.96	18.18
36^a^	76	55	NT	NT	NT	NT	NT

Treatment and management of ICP

To mitigate perinatal risks, comprehensive guidelines have been established covering fetal and biochemical surveillance, pharmacological interventions, and the timing of birth (see Table 2). Pharmacological strategies primarily aim to alleviate symptoms and facilitate timely birth to safeguard the fetus. Ursodeoxycholic acid, commonly known as ursodiol or by the brand name Actigall, is the preferred initial treatment for ICP and is deemed safe for both the mother and fetus [11]. It not only reduces itching and bile acid levels but is also the only treatment shown to lower perinatal mortality. Ursodiol treatment can commence based on clinical symptoms such as itching without a rash, even if elevated bile acids and AST/ALT levels are not initially present. The starting dosage is typically 300 mg orally two to three times daily or 500 mg twice daily, with an initial dose ranging from 10 to 15 mg/kg per day, divided into two to three doses. This can be increased up to a maximum of 21 mg/kg per day to reduce itching further [13]. Ursodiol helps increase the solubility of bile acids, aiding their transfer from the liver to the gallbladder and reducing their adverse effects [15]. However, symptom relief may take one to two weeks, and a decrease in bile acids may take three to four weeks [16]. Possible side effects can include nausea, dizziness, and stomach upset. The effectiveness of ursodiol in improving adverse fetal outcomes remains a subject of debate [5]. Studies like those by Kong et al. have shown reductions in bile acid levels and associated decreases in preterm labor and fetal distress after weeks of treatment [16]. By contrast, the research by Walker et al. has found no definitive proof that ursodiol mitigates fetal distress or demise [15].

**Table 2:** Recommendation on fetus birth according to the total bile acid by the Society for Maternal-Fetal Medicine Reference: [13]

Total bile acid (nmol/mL)	Recommended timing of birth
<40	36-39 W; later end of the range is reasonable.
40-99	36-39 W; earlier end of the range should be considered.
≥100	36 W; if intense pruritus persists and is unrelieved with medication, prior history of ICP with fetal demise at <36 weeks gestation, or acute or preexisting liver disease with deteriorating liver function then consider birth at 34-36 weeks.

For diagnosed cases, outpatient fetal surveillance might include serial nonstress tests. Given the link between high bile acid levels and poor perinatal outcomes, regular bile acid testing is advised. Fetal monitoring through ultrasound may also be recommended on an individual basis. The highest bile acid levels observed at any point during the pregnancy should guide management decisions. Due to the strong correlation between total bile acids and perinatal risk, inducing labor and early delivery are often recommended as potentially the only measures to prevent fetal demise [13]. Puljic et al. indicate that the risk of stillbirth increases between 34 and 40 weeks gestation with ICP, and facilitating delivery at 36 weeks gestation can reduce the risk of fetal death in ICP-complicated pregnancies compared to waiting for spontaneous labor [17]. Di Mascio et al. suggest that considering delivery at 35-36 weeks is the most appropriate option when the total bile acids exceed 100 nmol/L [14]. The Society for Maternal-Fetal Medicine also provides guidelines on the best gestational ages for delivery to minimize fetal risk (see Table 2).

Despite these measures, early induction of labor and birth do not guarantee fetal well-being in ICP-complicated pregnancies, and the risks associated with early induction should be discussed during patient counseling. Ultimately, patient counseling and collaborative decision-making play crucial roles in managing ICP-complicated pregnancies. Bile acid levels and symptoms of ICP typically subside within four to six weeks postpartum. If improvement is not observed, co-management with a hepatologist may be necessary to address potential underlying conditions (e.g., cardiovascular and hepatobiliary disease). A referral to a hepatologist is advisable if bile acid levels remain abnormal six weeks post-delivery.

Psychological implications

Receiving a diagnosis of a "rare condition" during pregnancy can be startling and overwhelming. The diagnosis and symptoms of ICP often raise more questions than answers due to outdated and inconsistent guidelines. Many expectant mothers grappling with ICP find themselves asking, "Why did this happen to me?" without clear answers. Researchers have identified certain risk factors, such as mutations in the adenosine triphosphate-binding cassette genes ABCB4 and ABCB11 [18] and a previous history of ICP. However, these did not apply in my situation. The psychological effects of ICP are not well-documented, yet I have experienced them personally and observed them in others through virtual and social media support groups. The psychological burden includes, but is not limited to, the relentless itching, disrupted sleep from the discomfort, the anxiety of awaiting a diagnosis, the stress of potentially needing an early delivery, the risks associated with premature birth, the potential long-term health impacts on the child, and the profound grief experienced by those who have lost a baby due to ICP.

Implications for the baby

Premature birth can heighten specific risks, including respiratory distress syndrome and difficulties with feeding. These risks are elevated for infants born to mothers with ICP, even if they reach full-term [19]. Papacleovoulou et al. have established a link between maternal ICP and an increased likelihood of metabolic diseases in the offspring [20]. At present, there are no confirmed additional long-term complications in children born to mothers with ICP.

## Conclusions

Early onset of ICP in the first or early second trimester, particularly among ethnic minorities such as South Asians, who have lower prevalence rates for ICP and are underrepresented in clinical studies, requires greater awareness among physicians. An epidemiological study focusing on ICP, which disproportionately impacts one of the fastest-growing populations in the United States, is urgently needed. Often, concerns about itching during pregnancy are dismissed, leaving mothers feeling anxious and unsupported during this vulnerable period. It is crucial to enhance awareness of ICP among friends, family, and medical professionals. Prompt serum bile acid testing and the provision of evidence-based treatments for all women with ICP are essential.

Furthermore, immediate improvements are needed to reduce the cost of ursodiol and enhance its accessibility. Given that, the medication can be very expensive, especially for those without health insurance or prescription coverage. Funding should also be extended to cover pharmacological treatments for all pregnant women, regardless of immigration status, to prevent perinatal morbidity and mortality effectively. The consequences of untreated ICP can be severe, potentially leading to fatal outcomes for the fetus.
